# Preoperative Cryogenic Neurolysis Trends Toward Reduced Severe Postoperative Pain in Patients Admitted to the Hospital After Total Knee Arthroplasty

**DOI:** 10.7759/cureus.101918

**Published:** 2026-01-20

**Authors:** Jorge Perera, Robert Wood, Jacqueline Krumrey

**Affiliations:** 1 Orthopedics, Good Samaritan Regional Medical Center, Corvallis, USA; 2 Orthopedics and Traumatology, Good Samaritan Regional Medical Center, Corvallis, USA

**Keywords:** multimodal pain control, post operative pain control, total joint arthroplasty, total knee replacement postoperative recovery, total knee replacement (tkr)

## Abstract

Introduction

Cryogenic neurolysis is an emerging conservative intervention for knee pain. It involves the percutaneous application of low temperatures to peripheral nerves to produce a long-lasting nerve blockade. Although commonly used conservative treatments for knee osteoarthritis provide temporary relief and carry risks, cryogenic neurolysis may offer longer-term pain control with minimal complications. This study examined the trends of preoperative cryogenic neurolysis and its role in reducing postoperative pain and opioid consumption in patients undergoing total knee arthroplasty (TKA).

Methods

We conducted a retrospective observational study of all primary TKA patients treated by a single surgeon between February 21, 2023, and February 21, 2024. Patients were grouped based on whether they received cryogenic neurolysis within two weeks preoperatively. Outcomes included maximum visual analog scale (VAS) pain scores, morphine milligram equivalents (MME) administered on postoperative days zero and one, and opioid refills within six weeks. Statistical analyses included chi-square tests with effect sizes, two-sample t-tests, and Wilcoxon rank-sum tests.

Results

A total of 168 patients were included (92 cryogenic neurolysis; 76 controls). On postoperative day zero, 24 of 92 patients (26.1%) in the cryogenic neurolysis group and 24 of 76 patients (31.6%) in the control group reported severe pain (VAS ≥7), a non-significant difference (χ²(1, N = 168) = 0.62, p = 0.43, Cramer's V = 0.06). Among the subset of 128 overnight patients, 10 of 54 (18.5%) in the cryogenic neurolysis group and 19 of 74 (25.7%) in the control group reported severe pain on postoperative day one, also non-significant (χ²(1, N = 128) = 0.91, p = 0.34, Cramer's V = 0.08). No significant differences were found in inpatient MME consumption. Opioid refills were similar between groups, with 49 of 92 patients (53.3%) in the cryogenic neurolysis group and 43 of 76 patients (56.6%) in the control group receiving at least one refill (χ²(1, N = 168) = 0.18, p = 0.67, Cramer's V = 0.03).

Conclusions

Preoperative cryogenic neurolysis was not associated with statistically significant reductions in postoperative pain scores, inpatient MME usage, or opioid refills. However, a lower percentage of patients who received cryogenic neurolysis reported severe postoperative day zero and one pain, suggesting a possible early clinical benefit. Although trends favored the cryogenic neurolysis group, larger prospective studies are needed to better evaluate its role in multimodal pain management for TKA patients.

## Introduction

Cryogenic neurolysis represents an evolving conservative intervention for knee pain in the orthopedic field [1]. The intervention involves percutaneous application of low temperatures (-20°C to -100°C) to the peripheral nerves of the knee, which induces Wallerian degeneration to provide a long-term nerve blockade [2]. In common practice, the conservative treatment of osteoarthritic knee pain involves non-steroidal anti-inflammatory drugs (NSAIDs), bracing, physical therapy, opioids, intraarticular corticosteroid injections, and viscosupplementation. However, while these interventions can provide some pain relief, many do not provide long-term pain control, and many are associated with risks and complications [3]. Cryogenic neurolysis addresses these concerns as it has the potential to provide long-term pain control with minimal risks [1, 4]. A double-blind, multicenter randomized control trial demonstrated that cryogenic neurolysis significantly reduced osteoarthritic knee pain for 90 days compared to those who received sham treatment [1, 4]. Moreover, this and other studies demonstrate that cryogenic neurolysis's complication profile is mild in severity, transient, and does not require intervention. The most common complications are bruising, numbness, redness, swelling, and local tenderness, and there is minimal risk to surrounding tissue structures with this technique [1, 4]. In our cohort, no major complications were observed within the postoperative follow-up time frame. 

In addition to serving as a conservative treatment for osteoarthritic knee pain, cryogenic neurolysis can also be utilized to minimize total knee arthroplasty (TKA) postoperative surgical pain [1, 5, 6]. This is important because one of the most significant indicators of patient dissatisfaction following TKA is postoperative pain [6]. Furthermore, critical rehabilitation occurs in the postoperative weeks, and adequate pain control is needed to optimize the range of motion and strength [7]. Opioids are commonly used for this perioperative pain control, but opioids can be fraught with complications and drug dependence [8-10]. These detrimental effects include, but are not limited to, decreased respiratory drive, falls, altered mental status, and constipation [11]. In addition, a considerable number of TKA patients become opioid dependent or long-term opioid users following surgery. Additional complications associated with opioids, aside from those noted in the immediate postoperative period, include increased rates of prosthetic joint infection, patient dissatisfaction, and repeat surgery on the ipsilateral extremity [12].

The adjunct of perioperative cryogenic neurolysis for TKA patients has been shown to reduce opioid administration, pain scores, and hospital length of stay [13-15]. However, there remains a paucity of research detailing the efficacy of cryogenic neurolysis in TKA pain control; a scoping review published in 2023 only yielded nine articles on this topic [1]. In this study, we sought to add to the literature and validate previous research regarding the efficacy of cryogenic neurolysis in TKA pain control. We hypothesize that undergoing cryogenic neurolysis is associated with lower maximum pain scores, fewer opioid refill prescriptions, and less morphine milligram equivalents administered.

## Materials and methods

Study setting

We performed a retrospective cohort study of patients who underwent a primary TKA with a single board-certified surgeon at a level two trauma center, Samaritan Albany General Hospital, in Albany, Oregon, USA. At the participating institution, all primary TKA patients are seen at two weeks pre-operation. At this visit, patients are given the opportunity to receive outpatient cryogenic neurolysis prior to their surgical procedure. 

All cryogenic neurolysis procedures were performed by a fellowship-trained primary care sports medicine physician using real-time ultrasound guidance to identify and treat the anterior femoral cutaneous nerve (AFCN) and the infrapatellar branch of the saphenous nerve (ISN/IPBSN) along planned treatment lines. Treatments were delivered with the iovera system (Gen 2; Pacira CryoTech, Tampa, Florida) using a closed-end needle smart tip selected by target depth. The device uses pressurized liquid nitrous oxide (N₂O) with delivery from a cylinder at >850 psi, achieving focused subdermal cooling below -20 °C with an absolute minimum needle cooling center temperature listed as -88 °C. Per manufacturer guidance, sequential applications were performed with overlap by one insertion site; expected cooled region dimensions in agarose gel are: width 6.3 mm, height 6.6 mm, skin-warmer-to-cooling-center 7.9 mm (33-second cycle), and: 15.5 mm × 7.5 mm (60-second cycle), noting that size may vary with patient anatomy/physiology. All this information can be gathered on the iovera website and instruction manual.

All patients within this cohort underwent spinal anesthesia and were injected intraoperatively with bupivacaine around the incision site in a standardized fashion. Postoperative patients receive standard in-hospital care, including a standard starting dose of 5 or 10 mg of oxycodone every four to six hours as needed on postoperative day zero. Patients who are cleared by physical therapy and deemed safe for discharge, as well as those who experience no complications after anesthesia, are discharged on postoperative day zero. Patients who are not cleared by physical therapy, experience anesthesia complications, have difficulties with oral intake or voiding postoperatively, or have prolonged alterations in vital signs are placed on observation overnight at the hospital. All patients are discharged with a prescription for 42 pills of 7.5 mg hydrocodone as well as multimodal pain control, including Celebrex and gabapentin. Patients follow up on an outpatient basis with the primary surgeon at two, six, and 12-weeks post-operation and begin physical therapy on postoperative day zero. In addition to in-person appointments, medical staff attempt to reach out to patients via telephone on the first day after hospital discharge to discuss the patient’s pain control.

Study variables

Our institution's electronic health records were queried to identify all of a single surgeon's patients who underwent a primary TKA between February 2023 and February 2024. Demographic and clinical characteristics were extracted from patients' records. Patients' ambulatory visit records were queried to identify those who received cryogenic neurolysis (CPT code 6460) within two weeks prior to their TKA. Only index surgery was included for patients with more than one qualifying surgery in the study period.

The primary aim of our study was to investigate the efficacy of cryogenic neurolysis on post-operative pain control. Pain control was measured using the patient's maximum reported visual analog scale (VAS) pain score and total morphine milligram equivalents (MME) administered on postoperative days zero and one. VAS pain scores were documented on a 10-point scale every two hours by hospital staff. A score greater than or equal to 7.0 is classified as severe pain. Patients' total inpatient MMEs were extracted for each postoperative day that patients were admitted. In addition to inpatient pain control, we aimed to evaluate if patients had adequate outpatient pain control over the entire six-week post-operative period. Inadequate outpatient pain control was defined as receiving one or more opioid (hydrocodone, oxycodone, tramadol) prescription refills sent within six weeks post-operation by the surgical team. Two weeks and six weeks are the routine follow-up schedule at our particular institution, and this is why this was used as the time table benchmark for this particular study. 

Statistical analysis

Patients were stratified into two cohorts: those who received pre-operative cryogenic neurolysis and those who did not. Patients' demographic and clinical characteristics were compared between strata using two-sample t-tests, Pearson's Chi-squared, and Fisher's exact tests. No statistically significant differences were observed in baseline characteristics; chi-square, Mann-Whitney U tests, and risk ratios were used to compare differences in pain-management. An alpha level of 0.05 was used for all statistical tests. 

## Results

We identified 179 total knee replacement surgeries performed by surgeon JR at the participating institution between February 2023 and February 2024. Eleven surgeries were non-index procedures and were excluded from analysis, resulting in a final sample size of 168 eligible primary TKA patients. The study population was predominantly female, with 103 of 168 patients (61.3%) identifying as female. The mean age of the cohort was 70.5 years. The mean operative time for both cohorts was 124 minutes, and 166 of 168 patients (98.8%) were discharged by postoperative day two. Among patients who received preoperative cryogenic neurolysis, 18 of 92 (19.6%) were discharged home the same day, 73 of 92 (79.3%) on postoperative day one, and 1 of 92 (1.1%) on postoperative day two. Among patients who did not receive cryogenic neurolysis, 22 of 76 (28.9%) were discharged home the same day, 53 of 76 (69.7%) on postoperative day one, and 1 of 76 (1.3%) on postoperative day two. Chronic opioid use before surgery was present in 17 of 168 patients (10.1%), and the proportion of chronic opioid users was similar between the cryogenic neurolysis group (8 of 92, 8.7%) and the control group (9 of 76, 11.8%). This demographic data stratified by patient group is represented below in Table 1. 

**Table 1:** Demographic characteristics of patients stratified by study group The p-values for mean age, operation time, and BMI were calculated with two-sample t-tests. Pearson's Chi-squared and Fisher's exact tests (if any cell size was <5) were used to calculate p-values for all other variables

	Did not receive preoperative cryogenic neurolysis (N=76)	Received preoperative cryogenic reurolysis (N=92)	p-value
Mean age at time of surgery (SD)	70.2 (9.06)	70.8 (8.41)	0.65
Sex			
Female	46 (60.5%)	57 (62.0%)	0.98
Male	30 (39.5%)	35 (38.0%)	
Race			
White or Caucasian	73 (96.1%)	88 (95.7%)	0.90
Other or Unknown	3 (3.9%)	4 (4.3%)	
Primary payer			
Commercial	19 (25.0%)	13 (14.1%)	0.07
Medicaid	8 (10.5%)	4 (4.3%)	
Medicare	48 (63.2%)	72 (78.3%)	
Other or Unknown	1 (1.3%)	3 (3.3%)	
History of diabetes			
None	63 (82.9%)	78 (84.8%)	0.90
History of diabetes	13 (17.1%)	14 (15.2%)	
Chronic opioid use before surgery			
No	67 (88.2%)	84 (91.3%)	0.68
Yes	9 (11.8%)	8 (8.7%)	
Mean BMI at time of surgery (SD)	32.9 (6.6)	32.3 (5.8)	0.57
Mean operation time, minutes (SD)	124 (16.6)	124 (17.0)	0.76
Day of discharge			
Postoperative day 0	22 (28.9%)	18 (19.6%)	0.30
Postoperative day 1	53 (69.7%)	73 (79.3%)	
Postoperative day 2	1 (1.3%)	1 (1.1%)	

**Figure 1:**
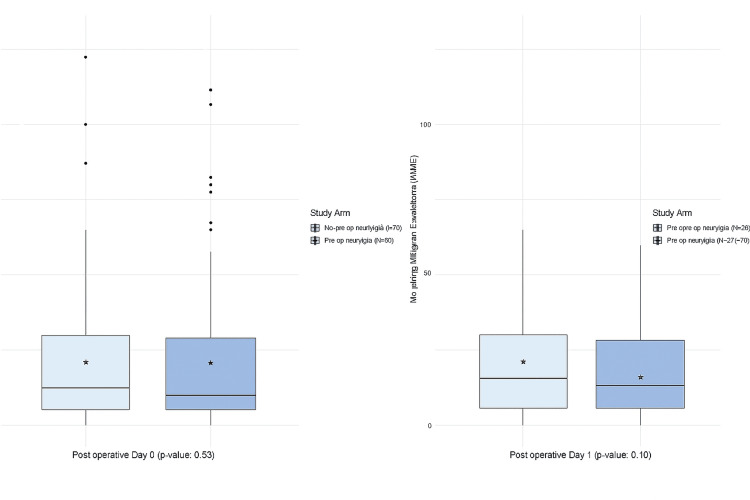
Distribution of morphine milligram equivalents (MME) stratified by study arm P-values calculated with the Mann-Whitney U test. Asterisks represent within-group means

A total of 126 study participants were hospitalized overnight and discharged on postoperative day one. Of these participants, an average of 12.5 and 14.1 morphine milligram equivalents was consumed on postoperative day zero and one, respectively. The distribution of MME consumption in the immediate postoperative period is shown in Figure 1. Patients who received preoperative cryogenic neurolysis consumed a median of 10.0 MME on postoperative day zero. In contrast, patients who did not receive preoperative cryogenic neurolysis consumed 12.5 MME on day zero. Among the subset of 126 patients hospitalized overnight, a median of 12.5 and 15.0 MME was consumed among preoperative cryogenic neurolysis patients and their control counterparts, respectively. No significant difference in MME consumption among study arms was identified on postoperative day zero or one.

**Figure 2:**
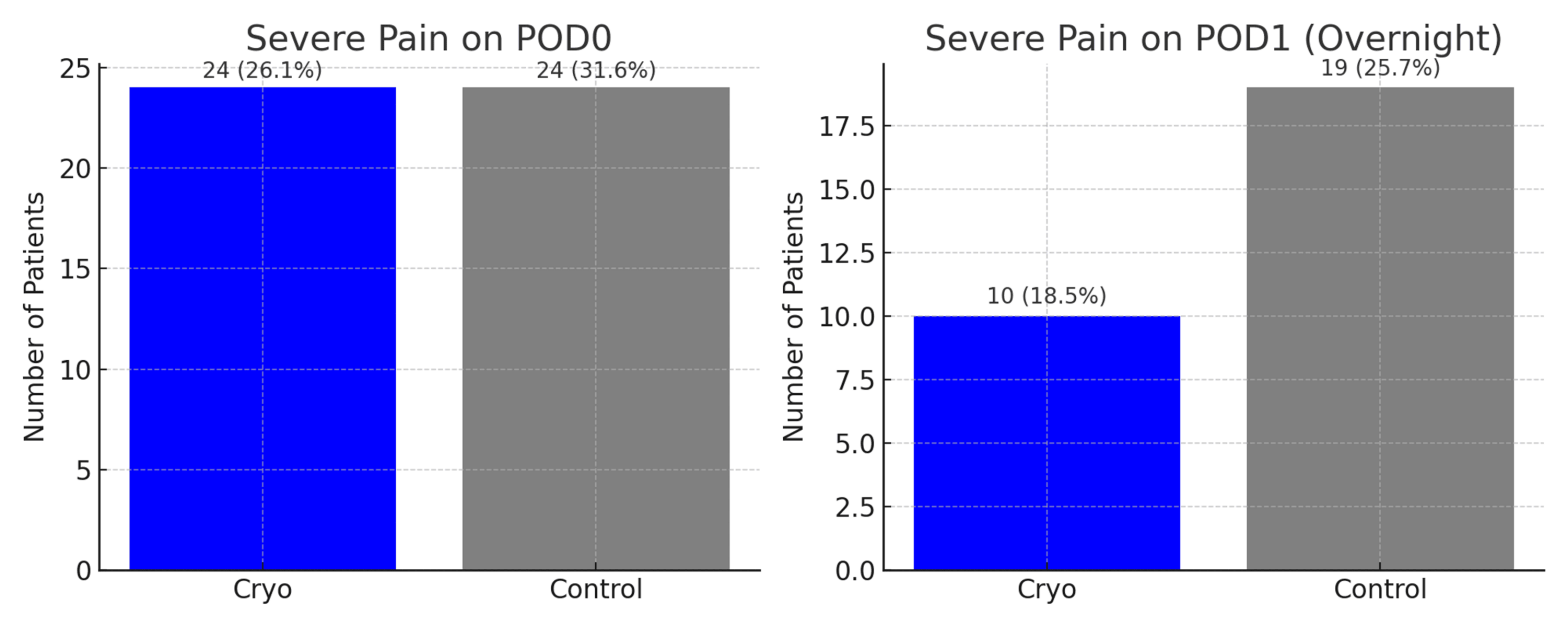
Proportion of study participants who reported severe pain (maximum VAS pain score ≥7) Values above each bar indicate the frequency (n) and percentage of patients in each group VAS - visual analog scale; POD - postoperative day

On postoperative day zero, 24 of 92 patients (26.1%) in the cryogenic neurolysis group and 24 of 76 patients (31.6%) in the control group reported severe pain (maximum VAS ≥ 7). This difference was not statistically significant (χ²(1, N = 168) = 0.62, p = 0.43, Cramer's V = 0.06). Pain scores remained stable overnight, with a medium maximum pain score of 5 reported in both groups on postoperative day one. On postoperative days zero and one, a lower proportion of patients who received preoperative cryogenic neurolysis reported severe pain (Figure 2). Patients who received preoperative cryogenic neurolysis had a 13.3% lower risk of reporting severe pain on postoperative day zero (RR: 0.87, 95% CI: 0.61 - 1.22, p-value: 0.43). Among patients admitted overnight, 10 of 54 patients (18.5%) in the cryogenic neurolysis group and 19 of 74 patients (25.7%) in the control group reported severe pain (maximum VAS ≥ 7) on postoperative day one. Although the proportion of patients with severe pain was lower in the cryogenic neurolysis group, this difference did not reach statistical significance (χ²(1, N = 128) = 0.91, p = 0.34, Cramer's V = 0.08).

**Figure 3:**
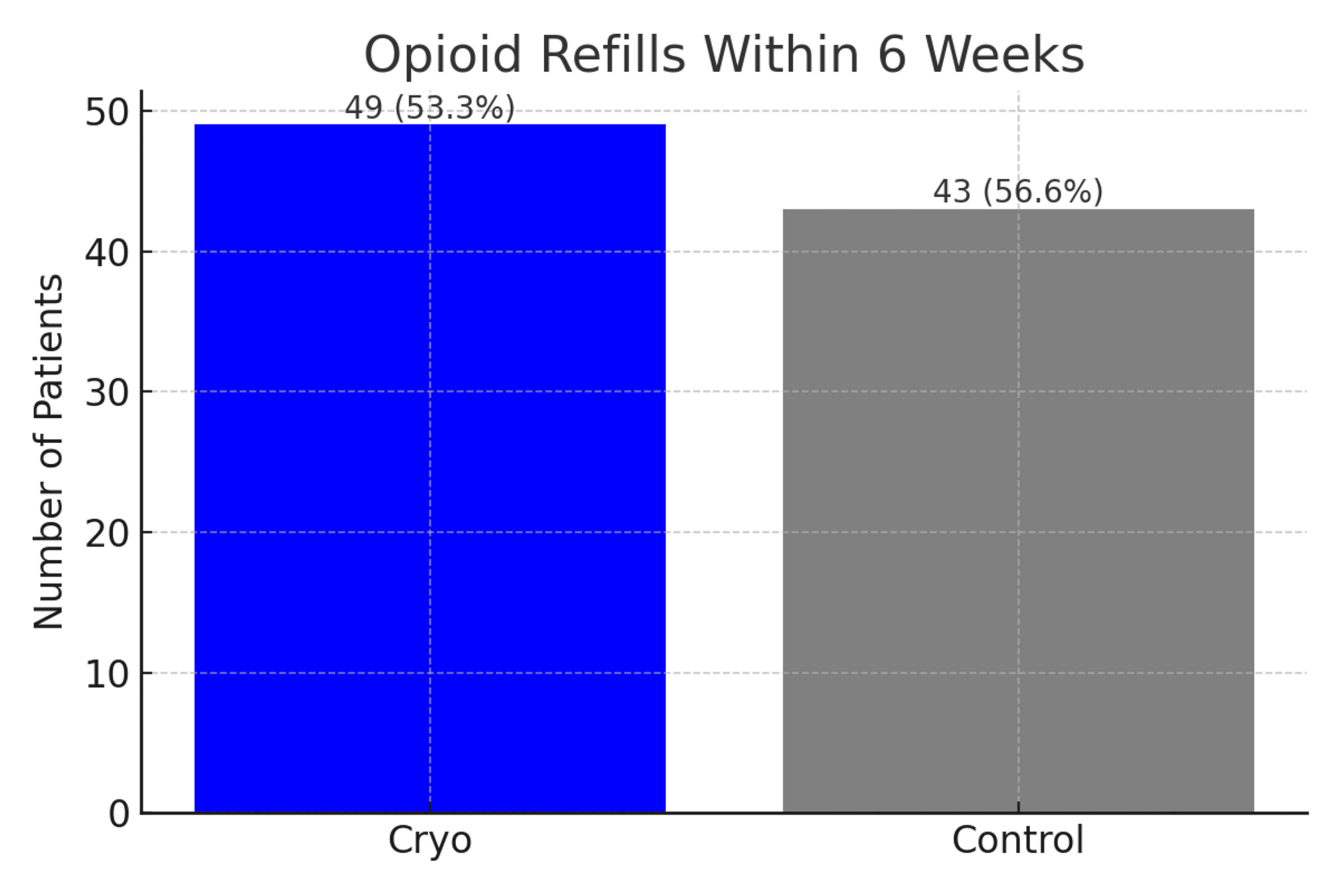
Proportion of study participants who received one or more opioid refills within six weeks Values above each bar indicate the frequency (n) and percentage of patients in each group

Overall, 92 of 168 patients (54.8%) received one or more opioid refills from the study team within six weeks post-operation. In the cryogenic neurolysis group, 49 of 92 patients (53.3%) received an opioid refill compared with 43 of 76 patients (56.6%) in the control group. There was no significant difference in refill rates between groups (χ²(1, N = 168) = 0.18, p = 0.67, Cramer's V = 0.03) (Figure 3). Importantly, no adverse events were systematically captured in the cryoneurolysis group during the entirety of the perioperative period observed. 

## Discussion

It is well understood that one of the most common reasons for TKA dissatisfaction is uncontrolled postoperative pain [16, 17]. This study aimed to add to the literature and to validate the research that already exists on the efficacy of cryogenic neurolysis as an alternative to analgesic therapy in the postoperative period of total knee arthroplasty. This study included comparing maximum pain scores and morphine milligram equivalents on postoperative days zero and one, along with comparing the likelihood of requesting a refill of prescription opioid drugs within the six-week postoperative period. On postoperative day zero, no differences in maximum pain scores were noted. Among patients admitted overnight, we observed a lower proportion of severe pain on postoperative day one in the cryogenic neurolysis group compared with controls, although this difference was not statistically significant (χ²(1, N = 128) = 0.91, p = 0.34, Cramer's V = 0.08). This trend towards significance for pain scores on postoperative day one coincides with our hypothesis that cryogenic neurolysis would result in lower pain scores. Our results also indicate positive, albeit non-significant, trends in MME consumption and opioid refills, also in favor of the cryogenic neurolysis group.

The extent of the effects of poorly controlled postoperative pain in TKA patients has been thoroughly examined and is now well understood. Due to this, multiple institutions have made it a priority to implement the most effective multimodal postoperative pain regimen available. In a multilevel model that examined total joint arthroplasty patients who received opioids only, versus two, three, or four modalities of analgesic therapy, the addition of pain control modes was associated with stepwise positive effects [18, 19]. This included fewer respiratory and gastrointestinal complications, a decrease in long-term opioid prescriptions, and a decrease in length of stay [6]. Of the same token, postoperative delirium has long been known to contribute to complications, including length of stay and increased hospital costs. In a prospective cohort study, which included 581 patients, it was found that those with high levels of postoperative pain and opiate use had the highest risk of developing delirium during their hospital stay [4, 20]. This further strengthens the argument for the need for a multimodal pain regimen following TKA, of which the addition of cryogenic neurolysis is a safe addition to include.

Regarding temperature-dependent neural effects, mild cooling preferentially blocks larger myelinated fibers earlier, while all fibers cease conducting around -20 °C, with colder exposures producing a reversible axonotmesis/ Wallerian degeneration profile (axon and myelin affected while the connective tissue scaffold is preserved); commonly cited effective cryogenic neurolysis targets are in the -60 to -100 °C range for reliable Wallerian degeneration while avoiding permanent ablation [21], whereas substantially colder temperatures (< -140 °C) have been associated with risk of permanent morphologic change [2]. While the procedure itself may be associated with numbness and dysesthesia, it has been found that these symptoms are self-limiting and have no long-term effect on patient outcomes if the practitioner is adherent to the principles of cryogenic neurolysis stated above [8]. Due to its safety and efficacy, the inclusion of this analgesic alternative to a multimodal regimen may soon become the standard for TKA postoperative pain control.

This study has several limitations. This study is retrospective and observational, which introduces an inherent risk of unmeasured confounding. This includes participant baseline pain, preoperative opioid use, and comorbidities, all of which may have had a significant impact on the outcomes of our study. Additionally, the sub-analysis excluded patients who were discharged the same day, as well as the overall limited sample size, may limit the power of our statistical analyses and could have resulted in a type II error. In addition, this study exclusively examined pain control in the immediate postoperative period, with the only long-term outcome measuring opioid prescription refill at the six-week mark. Further and more extensive follow-up may provide more revealing data and open the window for additional research opportunities.

## Conclusions

Overall, we found that among patients who were admitted overnight after TKA, there were improved outcomes for patients on postoperative day one with better pain control. Additionally, patients in the cryogenic neurolysis group were less likely to receive an opioid refill in the immediate postoperative period; however, the results were not statistically significant. These results are promising for future research, including randomized controlled trials directed at decreasing longer-term opiate consumption in postoperative TKA patients.
